# Characterization of the Hemocytes in Larvae of *Protaetia brevitarsis seulensis*: Involvement of Granulocyte-Mediated Phagocytosis

**DOI:** 10.1371/journal.pone.0103620

**Published:** 2014-08-01

**Authors:** Hyojung Kwon, Kyeongrin Bang, Saeyoull Cho

**Affiliations:** Department of Applied Biology, College of Agriculture and Life Science, Environment Friendly Agriculture Center, Kangwon National University, Chuncheon, Republic of Korea; Uppsala University, Sweden

## Abstract

Hemocytes are key players in the immune response against pathogens in insects. However, the hemocyte types and their functions in the white-spotted flower chafers, *Protaetia brevitarsis seulensis* (Kolbe), are not known. In this study, we used various microscopes, molecular probes, and flow cytometric analyses to characterize the hemocytes in *P. brevitarsis seulensis*. The circulating hemocytes were classified based on their size, morphology, and dye-staining properties into six types, including granulocytes, plasmatocytes, oenocytoids, spherulocytes, prohemocytes, and adipohemocytes. The percentages of circulating hemocyte types were as follows: 13% granulocytes, 20% plasmatocytes, 1% oenocytoids, 5% spherulocytes, 17% prohemocytes, and 44% adipohemocytes. Next, we identified the professional phagocytes, granulocytes, which mediate encapsulation and phagocytosis of pathogens. The granulocytes were immunologically or morphologically activated and phagocytosed potentially hazardous substances in vivo. In addition, we showed that the phagocytosis by granulocytes is associated with autophagy, and that the activation of autophagy could be an efficient way to eliminate pathogens in this system. We also observed a high accumulation of autophagic vacuoles in activated granulocytes, which altered their shape and led to autophagic cell death. Finally, the granulocytes underwent mitotic division thus maintaining their number in vivo.

## Introduction

Insects lack an acquired immune system but their innate immune system comprising cellular and humoral responses is well developed [1], [2], [3]. The cellular responses are characterized by immune activation of insect blood cells (hemocytes). Activated hemocytes kill pathogens through the process of phagocytosis, encapsulation, and nodulation. By contrast, the humoral responses include synthesis of antimicrobial peptides (AMPs) and activation of the phenoloxidase (PO) cascade, and can be activated by several known cascade pathways, such as the NF-kβ pathway, Toll and immune deficiency (Imd) pathway, Janus kinase (JAK)/STAT transcription factors pathway, and enzymatic cascades that regulate melanin formation and clotting [4], [5], [6], [7], [8], [9], [10]. Which of these responses is triggered first upon pathogen invasion of the insect body cavity (hemocoel) is still debated as a matter of contention. Nevertheless, because hemocytes interact directly with invading pathogens, the cellular response develops faster than the humoral response.

Insect hemocytes were first described by Swammerdam, and have since been characterized and categorized based on their structure, functions, and staining or histochemical reactions [11]. The most common types of hemocytes include prohemocytes, plasmatocytes, granulocytes, spherulocytes, adipohemocytes, coagulocytes, and oenocytoids [12], [13], [14]. However, confusion still exists regarding hemocyte categorization because hemocyte types vary greatly depending on the insect species. For example, only three hemocyte types were found in mosquitoes and flies, whereas in *Antheraea mylitta*, six hemocyte types were found in adults and another two types (vermicytes and podocytes) were observed in larva stages [15]. In addition, five to six different hemocyte types were found in the coleopteran species, such as *Rhynchophorus ferrugineus*, *Chaetocarabus lefebvrei* Dejean 1826, *Harmonia axyridis*, and *Xanthogaleruca luteolaMull* [16], [17], [18], [19].

Among these hemocyte types, plasmatocytes and granulocytes are considered as key players in cell-mediated immunity, although it is likely that other hemocyte types interact with plasmatocytes and granulocytes and contribute towards the immune response. Thus, granulocytes are the most abundant cell type in mosquitoes and play a major role in the cellular immune response [20]. In flies, plasmatocytes are the professional immune cell type and account for ∼95% of circulating hemocytes [21]. Meanwhile, both granulocytes and plasmatocytes carry out immune functions associated with encapsulation and phagocytosis in most Lepidoptera and some Coleoptera [17], [22].

In mammals, granulocytes such as neutrophils, eosinophils, and basophils are key players in the inflammatory response [23]. Both insect plasmatocytes or granulocytes and mammalian granulocytes function similarly, by engulfing and killing pathogens [24].

The process of phagocytosis is generally classified into two categories, heterophagy and autophagy. Heterophagy is the process of phagocytic swallowing of one cell by another to form intracellular phagosomes, which subsequently fuse with endosomes and finally with lysosomes leading to degradation of foreign materials [25]. During the initial stages of phagocytosis, the cells rapidly change their shape and extend pseudopodia or filopodia that contact and surround foreign materials. Once engulfed into the cytoplasm, foreign materials are enveloped into double-membrane vacuoles and finally degraded by fusion with lysosomes. Autophagy is a process whereby the cell degrades its own cytoplasmic content, such as protein aggregates and unnecessary organelles [26], [27]. Autophagy was originally described as a starvation response of the cell and as a survival mechanism to maintain energy homeostasis [28], [29]. Recently, however, it was revealed that autophagy performs innate and adaptive immune effector functions by facilitating pathogen detection and mediating pathogen clearance [27], [30]. Thus, autophagy represents yet another line of cellular defense against microbial pathogens that invade the cell [25]. Autophagosomes are double or multimembranous vesicles generated from specialized regions of the endoplasmic reticulum (ER), whereas phagosomes originate from the plasma membrane and are double-membrane vesicles [31].

Autophagy is a key process for maintaining cellular homeostasis, while heterophagy is important in the cellular response against pathogens, and under specific circumstances, their functions overlap. For example, when phagocytosed bacteria survive in phagosomes, autophagy can be stimulated to act as a back-up system to fight off pathogens [25]. *Listeria monocytogenes*, *Francisella tularensis*, Group A *Streptococcus*, and *Shigella flexneri* evade the bactericidal functions of macrophages by puncturing the phagosomal membrane and escaping into the cytoplasm where they are wrapped by vacuoles labeled by the autophagosomal protein light chain 3 (LC3), which is essential for the extension of autophagic membranes [28], [32]. Although it was shown that autophagy related to immune defense can be induced by bacteria selectively, it is likely to be stimulated whenever cells are confronted with endogenous danger signals [25].

Constitutive activation of autophagy can lead to type II programmed cell death (type II PCD) characterized by the accumulation of autophagic vacuoles in the cytoplasm [26]. On the other hand, type I programmed cell death (type I PCD) or classical apoptosis is characterized by nuclear condensation and DNA fragmentation without autophagosome formation. Although there is extensive evidence showing that some morphological, biochemical, and molecular features of type I and type II PCD are distinct, the distinction between these two types of cell death is not always clear, since caspase-dependent autophagic cell death and autophagic cell death carrying nuclear condensation and DNA fragmentation have also been reported [26], [33], [34].

This research is mainly focused on the cellular immune response in white-spotted flower chafers, *Protaetia brevitarsis seulensis* (Kolbe). This insect was very easy to maintain under laboratory conditions, and manipulate during experiments to obtain sufficient number of hemocytes. Moreover, the average period of larva stage on the ground is about 50 days under constant conditions. We hypothesized that the cellular defense system in this insect is well developed. Here, we report a novel finding showing that the granulocytes in this insect are a pivotal player in cellular immune responses. We identified six hemocyte types based on their size, morphology, and dye-staining properties and examined the quantitative changes of hemocytes in the hemolymph following bacteria and yeast invasion of the hemocoel. The granulocytes were specialized to perform specific functions, such as phagocytosis and encapsulation, and carried out autophagy-related phagocytosis. In addition, the high accumulation of autophagic vacuoles in activated granulocytes altered their shape and led to cell death. The granulocytes maintained their number in vivo by undergoing mitotic division.

## Results

### Hemocyte morphology and quantitative changes

**Figure 1:**
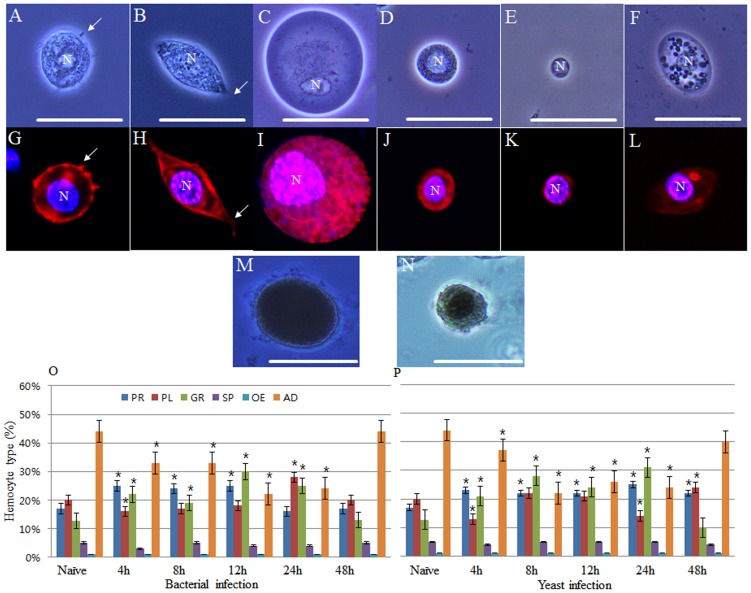
Images of hemocytes and average proportional distribution of circulating hemocytes in naïve and challenged larvae. Confocal images of hemocytes stained with DAPI (blue) for nuclei (N) and with filamentous actin (F-actin; red) for cytoskeleton visualization. Hemocytes were classified as granulocytes (A and G), plasmatocytes (B and H), oenocytoids (C and I), spherulocytes (D and J), prohemocytes (E and K), and adipohemocytes (F and L) on the basis of their size, morphology, and dye-staining properties. Pseudopodia or filopodia on the plasma membrane, are indicated by white arrow (A, B, G, and H). The panels M and N show melanized oenocytoids (M) and granulocyte (N). Hemocyte counting was performed by differential hemocyte count (DHC) and 35 larvae (11,235 hemocytes; 321 hemocytes per larva) were used to determine the percentage of the six circulating hemocyte types; five naïve larvae and three challenged larvae at each time point (4, 8, 12, 24, and 48 h h) post infection. Results are given as means and standard deviation. *(P<0.05). Challenged larvae were infected with *E*. *coli* (panel O) or *S*. *cerevisiae* (panel P) whereas naïve larvae were injected with sterile saline solution. PR, prohemocytes; PL, plasmatocytes; GR, granulocytes; SP, spherulocytes; OE, oenocytoids; AD, adipohemocytes. Scale bar = 20 µm.

Insect hemocyte classification is currently a matter of debate, however, we classified hemocytes based on their size, morphology, and dye-staining properties as previously described [12]–[14], [16]–[19], [20]–[22]. The circulating hemocytes in late instar larvae of *Protaetia brevitarsis seulensis* were classified based on their size, morphology, and dye-staining properties into six types, including granulocytes, plasmatocytes, oenocytoids, spherulocytes, prohemocytes, and adipohemocytes (Fig. 1A–L). As shown in Fig. 1A and G, the granulocytes (average width, 10.97 µm; average length, 14.87 µm, n = 30) were medium to large sized, round or slightly oval cells with many characteristically small granules in their cytoplasm. Their plasma membrane had a few very small pseudopodia or filopodia and their nuclei were round or elongated and relatively centrally located. After pathogen exposure, the granulocyte pseudopodia or filopodia transformed into considerably larger lobopodia-like or fan-like structures (fibroblastic-like or amoeba-like morphology). In addition, many of the small granules in the cytoplasm changed into comparatively larger sized polymorphic glittering vacuoles. The plasmatocytes (average width, 6.69 µm; average length, 22.46 µm, n = 30) had a typical spindle shape (Fig. 1B and 1H). Their nuclei were generally circular and located in the middle of the cell. Long pseudopodia or filopodia were observed on their plasma membrane. After exposure to pathogens, several longer filopodia were observed on their plasma membrane, but there were no sudden or rapid changes of shape and polymorphic vacuoles were not observed in their cytoplasm. In addition, while the granulocytes agglomerated together to form a large cluster around pathogens, the plasmatocytes did not.

The oenocytoids (average width, 18.82 µm; average length, 19.37 µm, n = 30) were scarce in the hemocoel, and had a round (fried egg-shaped) or sometimes irregular shape (Fig. 1C and 1I). The pseudopodia or filopodia were not found on the plasma membrane, which was optically very smooth. The nucleus was generally round and the cytoplasm was thick and slightly opaque. The spherulocytes (average width, 11.68 µm; average length, 11.82 µm, n = 30) were regularly spherical and medium sized with many small shinning granules in the cytoplasm (Fig. 1D and 1J). The live spherulocytes did not stain with 4′, 6-diamidino-2-phenylindole (DAPI) and fluorescently-conjugated phalloidin could not marking actin filaments but stained well after fixation in paraformaldehyde. The prohemocytes (average width, 6.6 µm; average length, 7.03 µm, n = 30) were the smallest round cells among the circulating hemocytes (Fig. 1E and 1K). The plasma membrane was optically smooth without any structures and their nuclei were large compared with their cell size, and located in the middle of the cell. The adipohemocytes (average width, 12.03 µm; average length, 21.53 µm, n = 30) were typically large oval cells with relatively large granules in the cytoplasm, and the plasma membrane was entirely smooth without any structures (Fig. 1F and 1L). The oenocytoids, spherulocytes, prohemocytes, and adipohemocytes did not change their shape after exposure to pathogens.

In insects, melanotic encapsulation of pathogen is an important part of the innate immune response and phenoloxidase (PO), one of the rate-limiting enzymes, is a key enzyme. To check which hemocytes show PO activity, we stained the hemocytes with dopamine dissolved in 35% alcohol as a substrate. PO activity was mainly detected in granulocytes and oenocytoids (Fig. 1M and 1N). Together, these data indicate that the granulocytes in this insect are a key cellular player in the defense against pathogens.

We next assessed the changes in relative percentages of hemocyte types in healthy larvae (naïve), which were injected with sterile saline solution and infected larvae (challenged), which were injected with bacteria (*Escherichia coli*) and fungi (*Saccharomyces cerevisiae*). The counting of hemocytes was performed by differential hemocyte counts (DHC) and 35 larvae (11,235 hemocytes) were used to calculate the percentage of circulating hemocyte types; five naïve larvae, and three challenged larvae at each time point (4, 8, 12, 24, and 48 h) post infection with bacteria and fungi. The percentages of circulating hemocyte types in naïve larvae were as follows: 13% granulocytes, 20% plasmatocytes, 1% oenocytoids, 5% spherulocytes, 17% prohemocytes, and 44% adipohemocytes (Fig. 1O). There was a significant (P<0.05; χ^2^) increase in the percentage of granulocytes at 12 h post infection with bacteria (30%) compared with that in naïve larvae (13%), which decreased (13%) at 48 h post infection (Fig. 1O). The percentage of plasmatocytes was increased at 24 h post infection (28%) compared with that in naïve larvae (20%), and then decreased (20%) at 48 h post infection. By contrast, the percentage of adipohemocytes was decreased at 24 h post infection (24%) compared with that in naïve larvae (44%), and then increased (44%) at 48 h post infection. However, we found no evidence that the adipohemocytes might have differentiated into granulocytes or plasmatocytes, suggesting that there is no direct relationship between these hemocyte types. The percentage of oenocytoids, prohemocytes, and spherulocytes was the same between naïve and challenged larvae (Fig. 1O). For larvae challenged with fungi, the quantitative changes of hemocytes were similar to those observed in larvae challenged with bacteria (Fig. 1P). During these experiments, the granulocytes changed their shape substantially and rapidly, compared with the other hemocyte types.

### Granulocytes as a pivotal player in cellular immune responses

**Figure 2:**
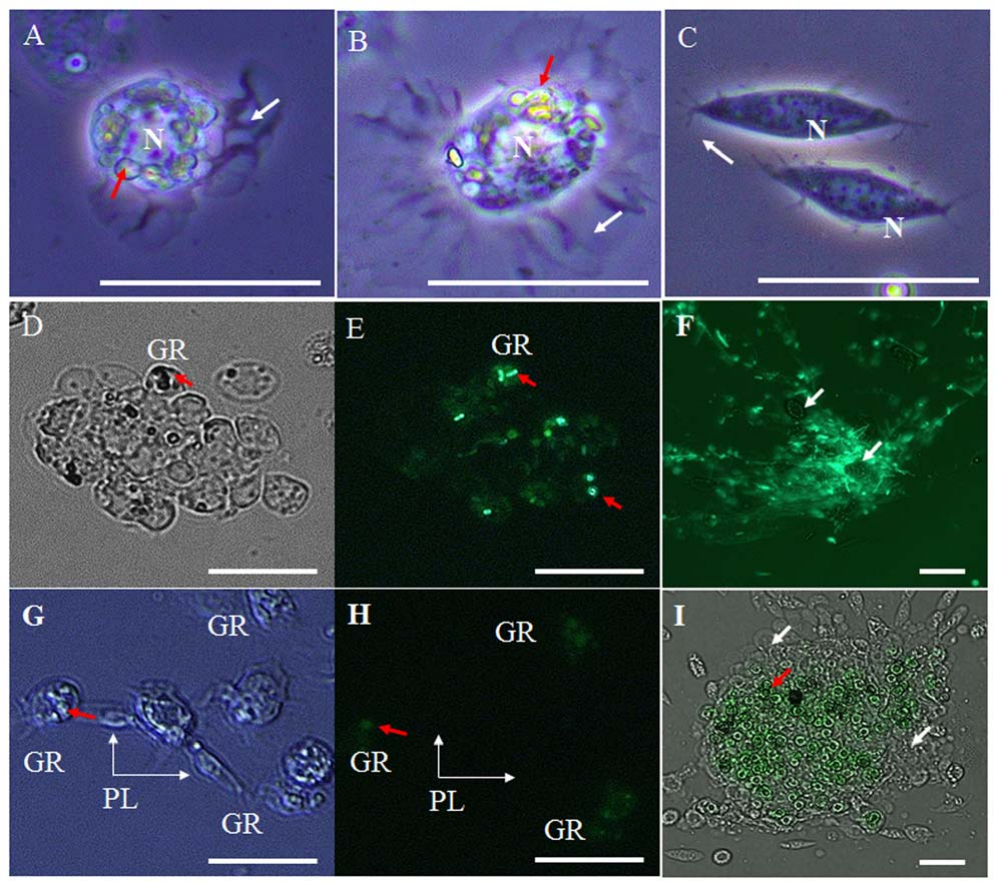
Images of immunologically activated granulocytes and encapsulation. (A and B) granulocytes changed their shape rapidly and generated large lobopodia-like, fan-like, or amoeba-like structures (indicated by white arrow). The highly polymorphic glittering vacuoles of variable size and round or irregular shape were closely packed in the granulocyte cytoplasm (indicated by red arrow). Although several filopodia were also generated in the plasmatocytes (C; indicated by white arrow), they did not undergo any sudden or rapid changes in shape and had no polymorphic vacuoles in their cytoplasm. With the exception of most granulocytes and some plasmatocytes, the other cell types showed no morphological changes compared to naïve larvae hemocytes (data not shown). (D and E) The granulocytes phagocytosed and engulfed GFP-expressing *E*. *coli* at 4 h post infection (GFP-expressing *E*. *coli* are indicated by red arrows). (G and H) Phagocytosis of GFP-expressing *S*. *cerevisiae* (indicated by red arrow) by GRs (indicated by short white arrow). Encapsulation by granulocytes at 12 h post bacterial (panel F; GFP-expressing *E*. *coli* phagocytosed by granulocytes are indicated by white arrows) and yeast infection (panel I; GFP-expressing *S*. *cerevisiae* are indicated by red arrow, and granulocytes are indicated by white arrow). GR, granulocytes; PL, plasmatocytes. N, nuclei, Scale bar = 20 µm.

To confirm whether the morphological changes in granulocytes were induced by cellular immune activity, such as phagocytosis, we conducted in vivo assays by injecting the larvae with GFP expressing *E*. *coli* (0.5–1 µm diameter), *S*. *cerevisiae* (5–10 µm diameter), or 1 µm carboxylate-modified polystyrene latex beads. A DIC light and fluorescence microscope analysis showed that the granulocytes were the main phagocytes in this insect. At 12 h post bacterial infection, over 90% of granulocytes in vivo rapidly changed their shape, and showed large lobopodia-like structures and fan-like (amoeba-like; indicated by white arrow) structures (Fig. 2A and 2B). In addition, highly polymorphic glittering vacuoles of variable size and round or irregular shape were closely packed in the granulocytes cytoplasm and underwent a remarkable expansion during the time course of infection (indicated by red arrow). Although approximately 5% of plasmatocytes generated several filopodia structures and occasionally expanded in size after exposure to pathogens, there were no sudden or rapid changes in their shape and no polymorphic vacuoles in their cytoplasm (Fig. 2C). With the exception of most granulocytes and some plasmatocytes, the other cell types did not change their morphology compared to naïve larvae hemocytes. The GFP-expressing *E*. *coli* and *S*. *cerevisiae* were captured inside the granulocytes, which showed conspicuous phagocytic activity (Fig. 2D through 2I). At 4 h post infection, the granulocytes engulfed the GFP-expressing *E*. *coli* (Fig. 2D and 2E; indicated by red arrows) and *S*. *cerevisiae* (Fig. 2G and 2H; indicated by red arrow). Very occasionally, pathogens were captured by plasmatocytes but not by the other hemocyte types. Some clusters composed of granulocytes and *E*. *coli* or granulocytes and *S*. *cerevisiae* were observed at 4 h post infection and considerably bigger clusters (encapsulation) could be seen at 12 h post infection (Fig. 2F and 2I). The plasmatocytes were partly involved in the encapsulation of pathogens since we occasionally observed a small number of plasmatocytes in these clusters, whereas oenocytoids, prohemocytes, spherulocytes, and adipohemocytes did not participate in the encapsulation process.

**Figure 3:**
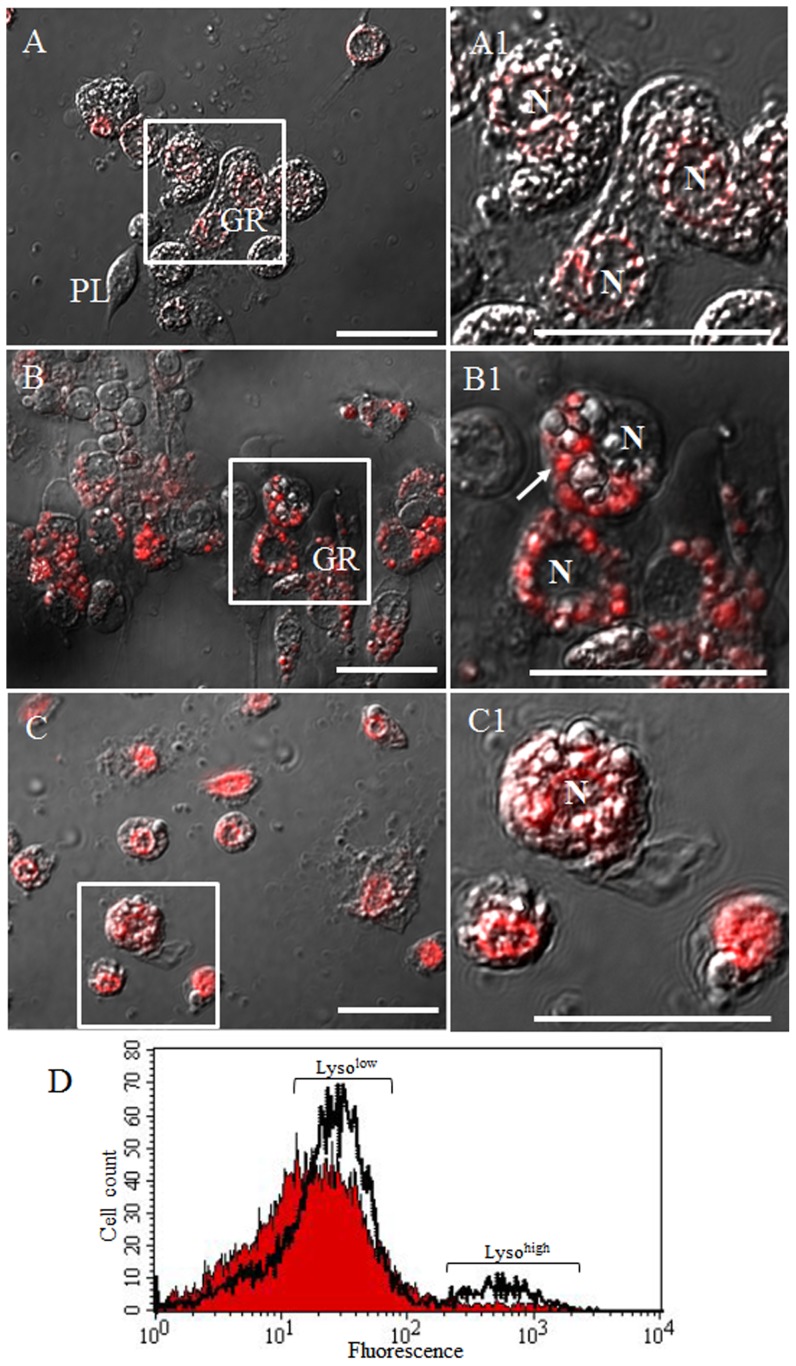
LysoTracker Red labeling of lysosomes in granulocytes and flow cytometric analysis after non-florescence carboxylate-modified polystyrene latex beads injection. (A and A1) 0 h post injection, (B and B1) 12 h post injection, and (C and C1) 48 h post injection, and (D) flow cytometric analysis at 12 h post injection. Data shows representative histogram overlays (filled histogram, 0 h post injection h post injection; and transparent histogram, 12 h post injection h post injection). The red filled histogram and transparent histogram indicate naïve and challenged larvae, respectively. Based on the red fluorescence intensity, two peaks were identified, Lyso^low^ and Lyso^high^. In the Lyso^low^ region, approximately 66.68% of naïve were increased to 77.44% at 12 h post infection. In addition, 1.93 h post infection. In addition, 1.93% of hemocytes of naïve were increased to 7.73% of hemocytes at 12 h post infection in the Lyso h post infection in the Lyso^high^ region. A1, B1, and C1 indicate a higher magnification of the regions in inset of panel A, B, and C. Over 90% of granulocytes were strongly stained by LysoTracker and the staining was especially prominent in their highly polymorphic vacuoles. GR, granulocytes; N, nuclei, Scale bar = 20 µm.

To examine whether the highly polymorphic large glittering vacuoles generated in the granulocytes were phagosomes, total hemocytes were stained with LysoTracker Red, which marks acidified compartments in cells after non-florescence carboxylate-modified polystyrene latex beads injection. LysoTracker Red faintly stained the granulocyte’s nucleus membrane at 0 h infection (Fig. 3A and 3A1). However, at 12 h post injection, over 90% of granulocytes showed prominent LysoTracker staining especially in the highly polymorphic vacuoles (Fig. 3B and 3B1). At 48 h post injection, approximately 50% of granulocytes showed strong staining in the highly polymorphic vacuoles and the rest of granulocytes showed staining in the nucleus membrane (Fig. 3C and 3C1). Highly polymorphic glittering vacuoles in granulocytes were also generated after bacterial and yeast infection, and were stained strongly with LysoTracker Red (data not shown). To quantify the red-fluorescent signal in naïve and challenged larvae, whole hemocytes were analyzed by flow cytometry after staining with LysoTracker Red at 0 and 12 h post infection. The flow cytometric assay revealed an increase in red-fluorescent signal at 12 h post infection (Fig. 3D). There were two peaks (Lyso^low^ and Lyso^high^) according to the fluorescence intensity. In the Lyso^low^ region, approximately 66.68% of naïve were increased to 77.44% at 12 h post infection. In addition, 1.93% of hemocytes of naïve were increased to 7.73% of hemocytes at 12 h post infection in the Lyso^high^ region (Fig. 3D). These results indicated that the highly polymorphic large glittering vacuoles generated in granulocytes were phagosomal or lysosomal compartments involved in the process of pathogen degradation.

### Granulocyte phagocytosis is dependent on foreign particle size

**Figure 4:**
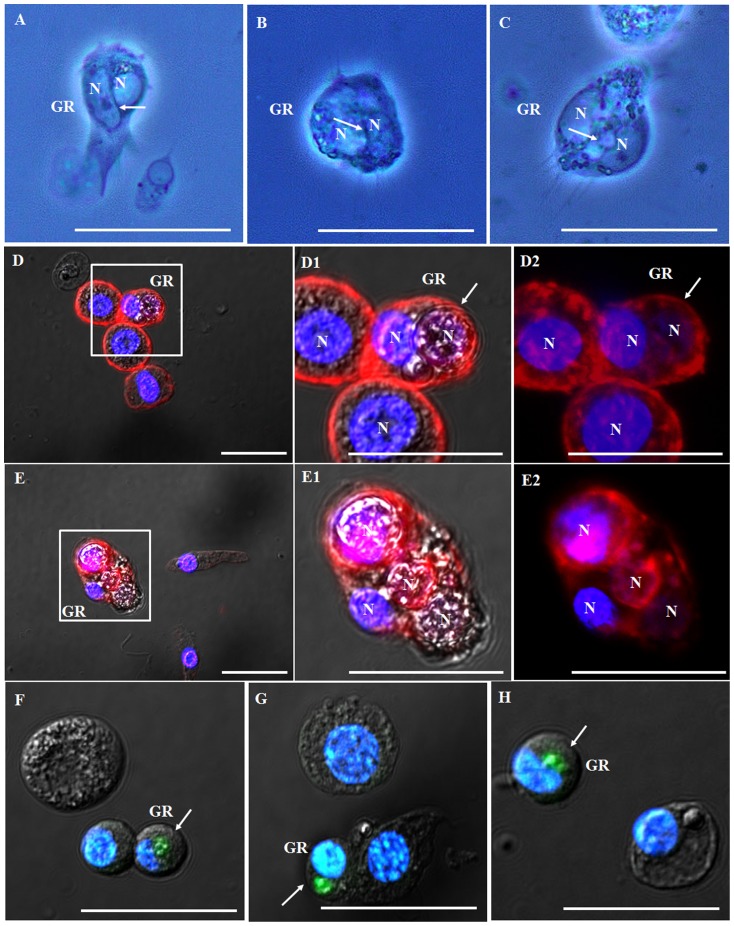
Complete internalization of large foreign particles by granulocytes. Hemocytes (average size 20.1 µm, n = 30) from the Stag Beetle *Lucanus maculifemoratus* (*Coleoptera*: Lucanidae) were injected into the hemocoel. At 12 h post injection, highly polymorphic glittering vacuoles were generated in the granulocytes. (A through C) The granulocytes engulfed and phagocytosed foreign cells and showed classic structures of phagocytosis (cell-in-cell invasion; a crescent-shaped nuclei) indicated by white arrow. (D through D2) Analysis by confocal microscopy demonstrated a complete internalization of hemocytes from *L*. *maculifemoratus* within the granulocytes. In some cases, one, or two, even three foreign hemocytes were phagocytized by the granulocytes (E through E2). D1, D2, E1, and E2 indicate a higher magnification of regions in inset of panel D and E. (F through H) GFP CellTracker dye labeled hemocytes (indicated by white arrow) from *L*. *maculifemoratus* were completely internalized by the granulocytes. GR, granulocytes; N, nuclei, Scale bar = 20 µm.

The carboxylate-modified polystyrene latex beads, bacteria, and yeast were of relatively small size compared to the size of granulocytes in this insect. To explore the granulocyte’s phagocytic capacities, we performed in vivo assays by injecting hemocytes (fairly bigger foreign materials: average size 20.09 µm, n = 30) from the Stag Beetle *Lucanus maculifemoratus* (Coleoptera: Lucanidae) into this insect. At 12 h post injection, highly polymorphic glittering vacuoles were generated in the granulocytes. Furthermore, we observed complete cell internalization by the granulocytes, which showed classic structures of phagocytosis such as crescent-shaped nuclei (Fig. 4A–C; indicated by white arrow). Analysis by confocal microscopy demonstrated the complete internalization of hemocytes from *L*. *maculifemoratus* within granulocytes (Fig. 4D–D2). In some cases, one, two, or even three foreign hemocytes were phagocytized by granulocytes (Fig. 4E–E2). For a more detailed examination, hemocytes from larvae of *L. maculifemoratus* were labeled with GFP CellTracker dyes and injected into the hemocoel of this insect. At 12 h post injection, the GFP labeled hemocytes were completely internalized within the granulocytes of *P*. *brevitarsis seulensis* (Fig. 4F–H; GFP labeled hemocytes are indicated by white arrow). Highly polymorphic glittering vacuoles were also generated in the granulocytes and stained with LysoTracker Red (data not shown).

**Figure 5:**
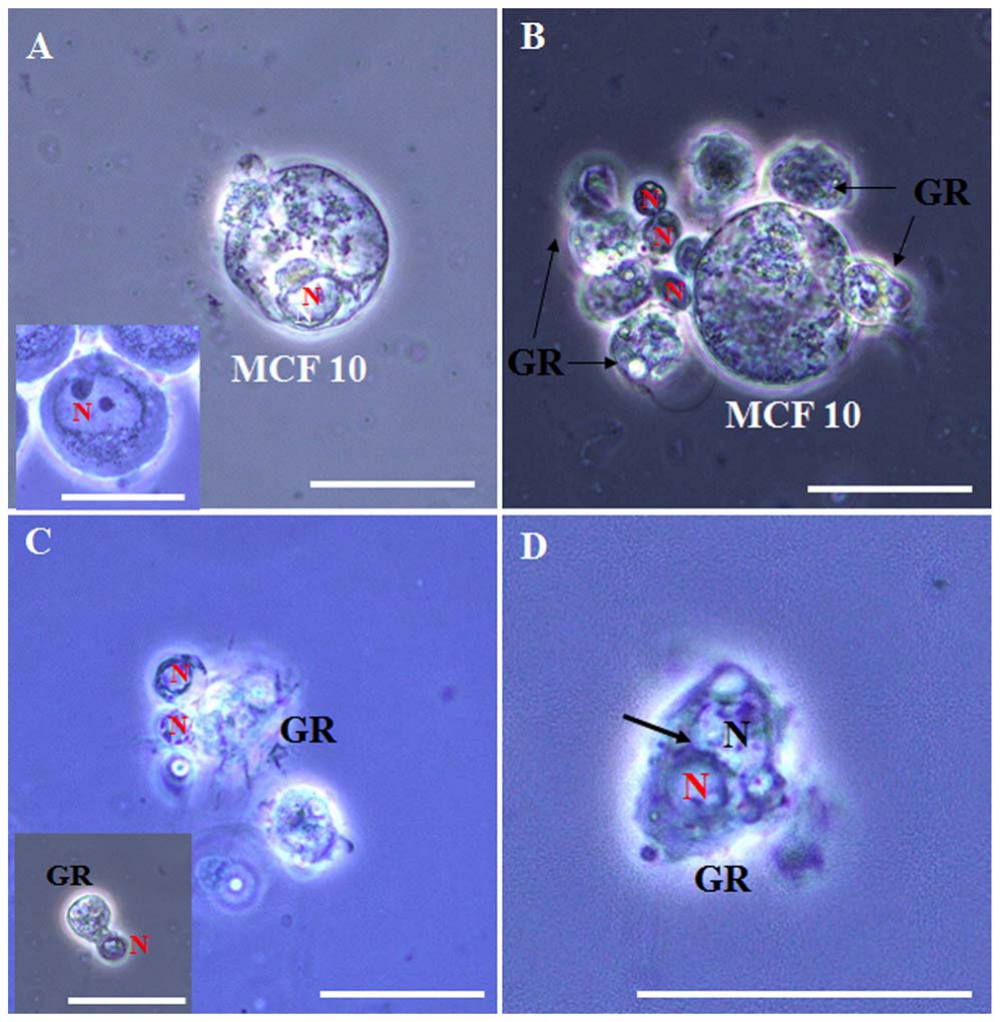
Granulocyte phagocytosis is dependent on foreign particle size. To examine the granulocyte’s phagocytic competence, human breast cells (MCF10; about two times bigger than granulocytes; average size ∼43 µm, n = 30) were injected into the hemocoel. (A) Autolysed MCF10 cells in the hemocoel; inset shows normal type of MCF10. (B) MCF10 nuclei indicated by red “N”). Shown is encapsulation by granulocytes, which cluster around the MCF10 cell. (C) Exposed nuclei from MCF10 agglomerated together and were phagocytosed by granulocytes; inset, granulocyte amoeba-like structure capturing MCF10 nuclei. (D) MCF10 nuclei (indicated by red “N”) were engulfed and phagocytosed by granulocytes, which showed classic structures of phagocytosis (cell-in-cell invasion; a crescent-shaped nuclei) indicated by black arrow. GR, granulocytes; N (red color), nuclei of MCF10 cells, N (black color), nuclei of granulocytes. Scale bar = 20 µm.

To further investigate the granulocyte’s phagocytic competence, we injected human breast cells (MCF10), which are about three times the size of granulocytes (average size ∼43 µm, n = 30), into the hemocoel. The granulocytes could not phagocytose the MCF10 cells, which were autolysed in the hemocoel (Fig. 5A; normal MCF10 (inset)). In many cases, however, we observed an encapsulation with the granulocytes clustering together and surrounding the MCF10 cells (Fig. 5B; MCF10’s nuclei indicated by red “N”). In some cases, we observed that many MCF10 nuclei were also agglomerated together and were phagocytosed by the granulocytes (Fig. 5C and 5D; inset shows granulocyte’s amoeba-like structure capturing MCF10’s nuclei). Collectively, these data demonstrate that phagocytosis by granulocytes is dependent on foreign particle size.

### LC3-associated phagocytosis (LAP) by Granulocytes

**Figure 6:**
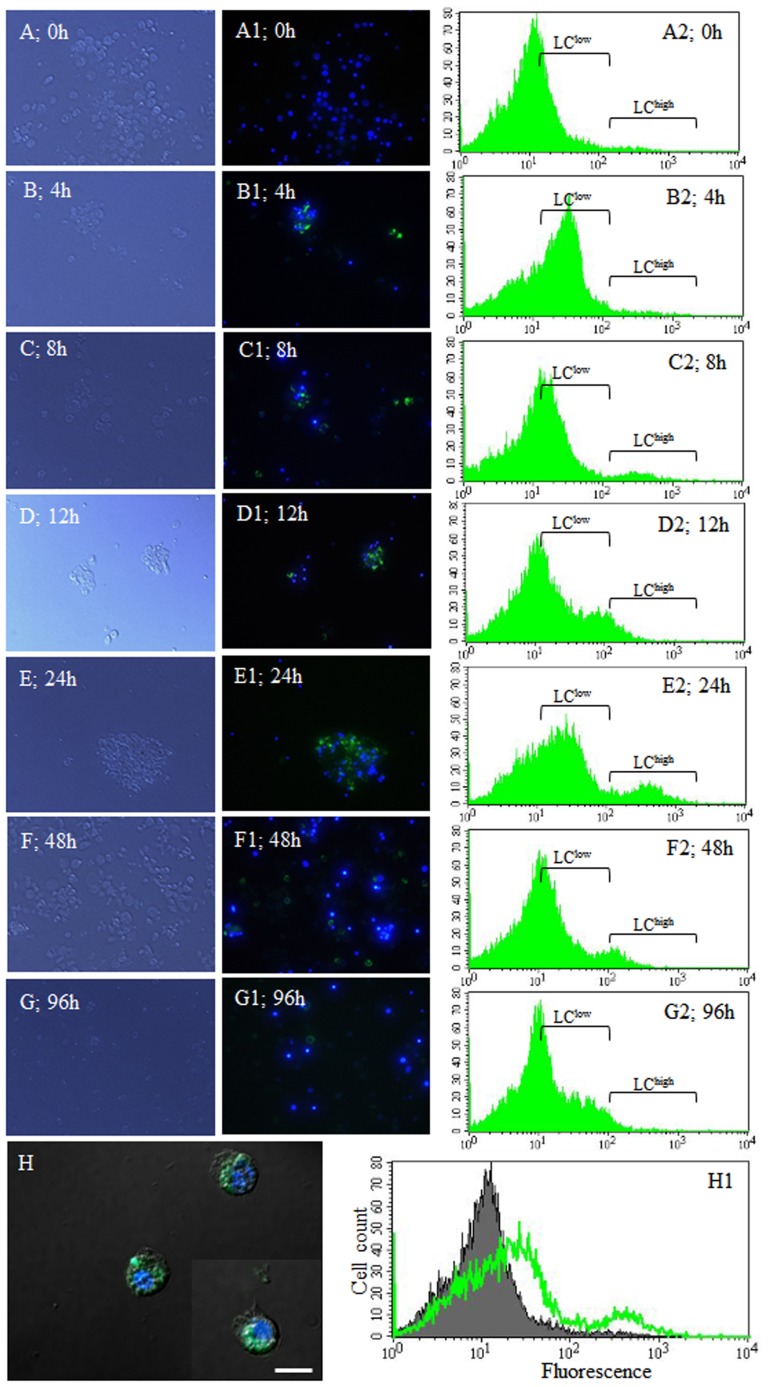
LC3-associated phagocytosis (LAP) by Granulocytes. Non-florescent carboxyl modified beads were injected into the hemocoel. GFP LC3 was used for detection of autophagosome formation in granulocytes at 0–96 h. (A–G) DIC microscope images and fluorescent microscope images (A1–G1) of granulocytes stained with DAPI and GFP-LC3; and flow cytometric analysis (A2–G2). (H) Confocal images of granulocytes stained with DAPI and GFP LC3 at 24 h post injection. (H1) Representative histogram overlay (filled histogram, 0 h post infection, A2 h post infection, A2; green transparent histogram, 24 h post infection, (E2). Based on the green fluorescence intensity, two peaks were identified, LC^low^ and LC^high^. (A2 and E2) 11.40% of granulocytes at 24 h post infection and 1.39 h post infection and 1.39% of granulocytes at 0 h post infection were in the LC h post infection were in the LC^high^ region, and approximately 10% of GFP-stained hemocytes were increased in the LC^low^ region at 24 h post infection. (F2 and G2) At 48 and 96 h post infection, LC3 accumulation in granulocytes decreased gradually. h post infection, LC3 accumulation in granulocytes decreased gradually.

Recently, it was reported that the microtubule-associated protein 1 light chain 3 alpha (LC3) is required for the efficient clearance of dead cells [36]. To determine whether the highly polymorphic glittering vacuoles generated in immunologically activated granulocytes were produced during the process of phagocytosis (phagosome) or LC3-associated phagocytosis (autophagosome), hemocytes were stained with GFP-LC3, a widely used marker of autophagosome formation, which is responsible for sequestering materials intended for delivery to lysosomes. At 0 h after non-florescent carboxyl modified beads injection, the granulocytes as well as other hemocyte types were negatively stained with GFP-LC3 (Fig. 6A and A1). However, we observed that the highly polymorphic vacuoles in the granulocyte cytoplasm showed LC3 accumulation as early as 4 h post injection (Fig. 6B and B1). At 24 h post injection, the LC3 accumulated highly in granulocyte vacuoles (Fig. 6E and E1) and decreased thereafter (Fig. 6F, 6F1, 6G, and 6G1), whereas the other hemocyte types were negatively stained throughout (data not shown). At 24 h post injection, the accumulation of GFP-LC3 in granulocyte’s vacuoles was examined by DAPI staining using a confocal microscope (Fig. 6H). In addition, flow cytometric analysis revealed that the increase in GFP-LC3 staining occurred at 24 h post injection and then, decreased gradually (Fig. 6A2–G2). Based on the green fluorescence intensity, there were two peaks (LC^low^ and LC^high^). 11.40% of granulocytes at 24 h post injection and 1.39% of granulocytes at 0 h post injection, were in the LC^high^ region (Fig. 6A2 and 6E2). Approximately 10% of GFP-stained hemocytes were increased in the LC^low^ region at 24 h post injection. Then, at 48 and 96 h post injection, LC3 accumulation in granulocytes decreased gradually (Fig. 6F2 and 6G2). Fig. 6H1 shows a representative histogram overlay (0 and 24 h post injection).

**Figure 7:**
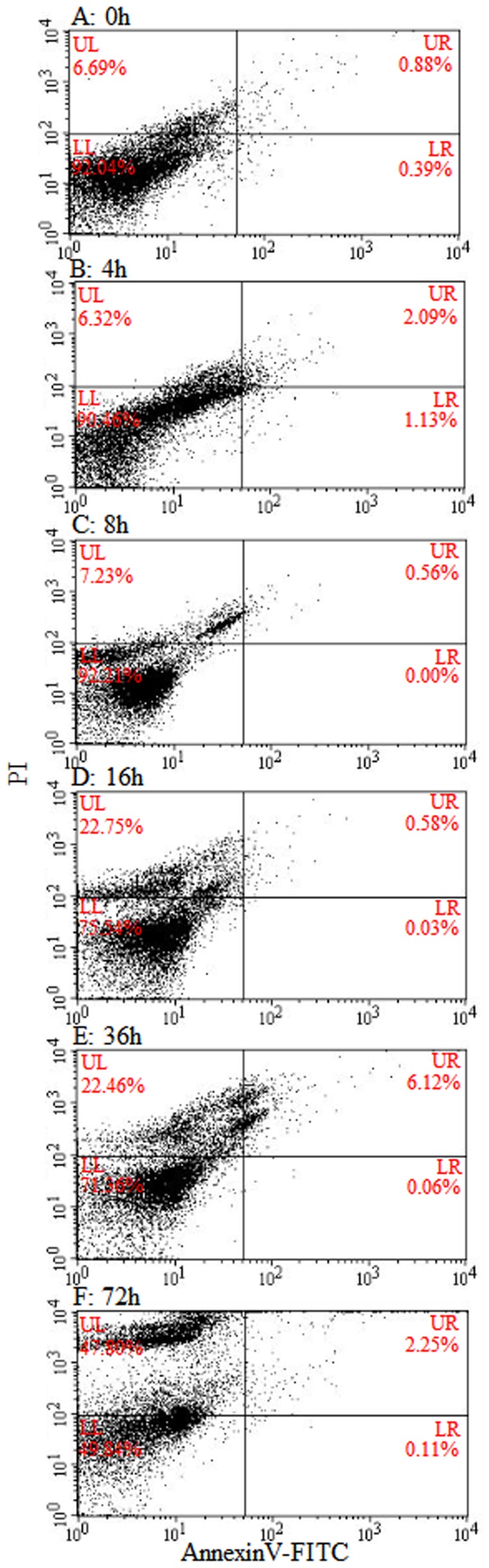
Flow cytometric analysis of hemocytes stained with Fluorescein isothiocyanate (FITC)-annexin/Propidium Iodide (PI). Non-florescent carboxylate-modified polystyrene latex beads were injected into the hemocoel at 0, 4, 8, 12, and 48 h. The percentage of PI negative and annexin h. The percentage of PI negative and annexin-V positive hemocytes (LR quadrant) was 0.39, 1.13, 0.00, 0.03, 0.06, and 0.11% at 0, 4, 8, 16, 36, and 72 h post injection, respectively. However, the percentage of PI h post injection, respectively. However, the percentage of PI-positive and annexin-V negative hemocytes (UL quadrant) increased from 6.69% at 0 h to 47.80 h to 47.80% at 72 h post injection. The percentage of PI h post injection. The percentage of PI-positive and annexin-V positive (UR quadrant) was 0.88% at 0 h and 2.25 h and 2.25% at 72 h post injection. h post injection.

The high accumulation of autophagic vacuoles in granulocytes altered their shape and induced cell death. After the injection of carboxylate-modified polystyrene latex beads, the hemocytes were stained with Fluorescein isothiocyanate (FITC) annexin-V/Propidium Iodide (PI) and analyzed by flow cytometry. The percentage of PI negative and annexin-V positive hemocytes was 0.39% at 0 h and 0.11% at 72 h post injection (Fig. 7; LR region). However, the percentage of PI-positive and annexin-V negative hemocytes increased from 6.69% at 0 h to 47.80% at 72 h post injection (Fig. 7; UL region). The percentage of PI-positive and annexin-V positive hemocytes was 0.88% at 0 h and 2.25% at 72 h post injection (Fig. 7). These results indicated that the high accumulation of autophagic vacuoles in hemocytes did not induce typical apoptotic cell death but autophagy or necrosis related cell death.

### Granulocyte mitosis induced by infection

**Figure 8:**
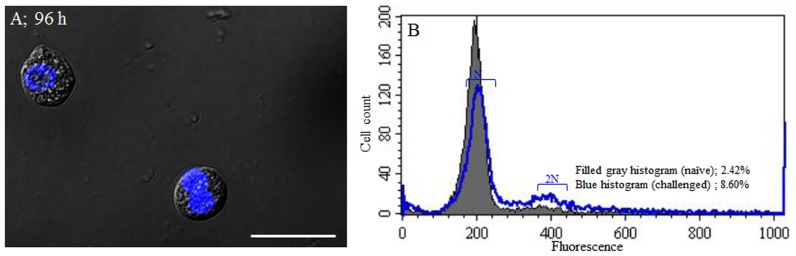
Granulocyte mitosis induced by non-florescent carboxyl modified beads injection into the hemocoel. (A) Analysis by DIC and fluorescent microscopes showed that the granulocytes were mitotically divided at 96 h post injection. (B) Flow cytometric analysis of granulocyte mitosis. Shown are representative histogram overlays: filled histogram (0 h injection h injection) and blue transparent histogram (96 h post injection h post injection). 2.42% of hemocytes in naïve larva were undergoing mitosis, compared to 8.60% of hemocytes in challenged larvae at 96 h post injection. h post injection.

Our findings of autophagy-related cell death, led us to investigate the mechanism by which granulocytes maintain their number in vivo. The rate of granulocytes mitosis in circulating cells was assessed by DAPI and fluorescently-conjugated phalloidin staining following carboxylate-modified polystyrene latex beads injection. At 96 h post injection, the DIC light and fluoresces microscope analysis showed that many granulocytes were mitotically divided (Fig. 8A). Flow cytometric analysis revealed that 2.42% of hemocytes in naïve larvae were undergoing mitosis, however, this mitotic index was increased to 8.60% at 96 h post injection (Fig. 8B). These results demonstrated that granulocyte mitosis might play a role in maintaining their number in vivo, independently of hematopoietic or progenitor cell types.

## Discussion

The results of this study clarify the role of granulocytes in the cellular immune response of *Protaetia brevitarsis seulensis* to pathogens. We show that granulocytes are the professional phagocytes in this insect and a key player in the fight against pathogens. We classified circulating hemocytes based on their size, morphology, function, and dye-staining properties, into six types, including granulocytes, plasmatocytes, oenocytoids, spherulocytes, prohemocytes, and adipohemocytes. To identify the professional phagocytes, we morphologically examined the six hemocyte types for the presence of pseudopodia or filopodia on their plasma membrane. Professional phagocytes can easily be identified in flies and mosquitos since these cells are the most abundant cell type and account for over 95% of circulating hemocytes [20], [21]. By contrast, in this insect, the granulocytes and plasmatocytes represented 13% and 20% of circulating hemocytes, respectively and thus, the identification of professional phagocytes was based solely on the presence of filopodia on their plasma membrane. As shown in Fig. 1B and 1H, plasmatocytes, which are the typical macrophage cell type in insects, had a typical spindle shape with several relatively long filopodia. On the other hand, the granulocytes had only a few small filopodia on the plasma membrane and were not abundant in the hemolymph (Fig. 1A, 1G, and 1M). Based on these findings, we initially classified the plasmatocytes as the only professional phagocyte in this insect.

However, regardless of which potentially hazardous substance was used as the infective agent, only the granulocytes became morphologically highly activated. As shown in Fig. 2, most granulocytes spread on glass slides extensively, and highly glittering polymorphic vacuoles of variable size and shape were generated in their cytoplasm. Most immune activated hemocytes in insects, including neutrophil granulocytes in humans, undergo morphological changes and generate networks of extracellular fibers. Neutrophils are professional phagocytes, which engulf and digest potentially hazardous substances, pathogens, and tumor cells, and are therefore classified as the first line of defense in the bloodstream [37]. The fan-like structures or networks of extracellular fibers in neutrophils, called neutrophil extracellular traps (NETs), consist of chromatin fibers and various proteins. Neutrophils use NETs to immobilize and kill pathogens [38]. As shown in Fig. 2, the sticky fiber webs generated in immune activated granulocytes were used for capturing yeast and bacteria, and for the formation of large granulocyte clusters needed for encapsulation. On the other hand, the plasmatocytes engulfed GFP-expressing bacteria and yeast only rarely, but did not undergo morphological changes or generate lysosome vacuoles in the cytoplasm. As shown in Fig. 3, the lysosomes or phagosomes were observed only in immunologically activated granulocytes, suggesting that granulocytes are the professional phagocytes in this insect. The only professional phagocytes in flies and mosquitos are plasmatocytes and granulocytes, respectively [20], [21]. In *M*. *unipuncta* plasmatocytes are not capable of phagocytosis and granulocytes are the main phagocytes. By contrast, the plasmatocytes are major phagocytes in the greater wax moth *Galleria mellonella* [13], [39]. Although several previous studies have shown that both plasmatocytes and granulocytes are involved in phagocytosis, our results identified granulocytes as the only professional phagocytes in larvae of *P*. *brevitarsis seulensis*.

The granulocytes in this insect engulfed potentially hazardous substances depending on the particle size. Furthermore, the granulocytes showed heterogeneous cell-in-cell phenomena and developed typical crescent-shaped nuclei as shown in Fig. 4. Originally, these phenomena were observed in many tumor cells but, as recently demonstrated, are not restricted to tumor cell lines. In mammals, the dying cells destined to be phagocytosed express or release various eat-me signals, find-me signals, and damage-associated molecular pattern molecule signals either directly or indirectly through bridging molecules [40]. The molecular mechanisms of phagocytosis by granulocytes remain to be elucidated [41].

While the process of phagocytosis involves the engulfment and digestion of one cell by another, in autophagy the cell degrades its own cytoplasmic content and unnecessary organelles [26], [27]. Recent studies have described the existence of autophagic-phagocytic hybrid processes, suggesting that autophagy has a dynamic relationship with phagocytosis [40]. Thus, pathogens in the phagosome can be eliminated by different pathways [42]. Our results showed that following infection the granulocytes cytoplasm was filled with highly polymorphic glittering vacuoles. As shown in Fig. 7, these vacuoles were strongly stained by LysoTracker Red, which marks acidified compartments (usually lysosome) in cells, and were fused with GFP-LC3. These results indicated that the uptake of potentially hazardous substances by granulocytes rapidly triggers the translocation of LC3 to the phagosome. Recently, several studies suggested that autophagy occurs in human and mouse neutrophils in both phagocytosis-independent and phagocytosis-dependent manner [43]. Autophagy helps to degrade intracellular hazardous substances, enhances pathogen killing, and facilitates the delivery of cytosolic proteins such as antimicrobial peptides [43]. Furthermore, autophagy can be stimulated when phagocytosed bacteria survive in the phagosome and can act as a back-up system to destroy pathogens [25]. We observed that vacuolized granulocytes were always stained with GFP-LC3 regardless of the foreign substances used. The (LC3)-associated phagocytosis occurred within 4 hours of pathogen engulfment and is likely constitutively induced in granulocytes. This result indicated that autophagy helps degrade intracellular hazardous substances and enhances pathogen killing.

Inflammation-associated autophagy could be associated with cell death mechanisms, which are enhanced during pathogen clearance. The large-scale accumulation of autophagosomes has been observed in the cytoplasm of many dying cells, including macrophages in various animals, and this autophagic cell death is known as cell death with autophagy rather than cell death by autophagy, and termed autophagic cell death (ACD) or type II programmed cell death (type II PCD) [26], [44], [45]. In addition, the formation of NETs by neutrophils may require autophagy and these neutrophils could undergo a necrotic form of cell death called NETosis [27], [43]. As shown in Fig. 3, the granulocytes were characterized by high accumulation of portions of the cytoplasm in autophagosomes giving the cell a characteristic vacuolated appearance [45]. In addition, sticky fiber webs reminiscent of NETs were observed on the granulocyte surface and, thus, the granulocytes were considered to be undergoing an autophagic-related necrotic form of cell death. As shown in Fig. 7, the percentage of both annexin-V positive and PI-positive granulocytes increased simultaneously during the time course of infection with no lag period [46]. These results indicated that the vacuolated granulocytes were undergoing ACD.

After delivery of immune stimuli, an increase in the number of hemocytes was observed in several insects [47]. In this insect, nuclear staining and flow cytometric analysis of hemocytes showed that granulocytes continuously undergo mitosis (proliferating by autonomous division) following ACD. Therefore, granulocyte mitosis could play a role in maintaining their number in vivo, independently of hematopoietic or progenitor cell types.

## Conclusions

In this study, we identified granulocytes as the professional phagocytes in *Protaetia brevitarsis seulensis*. The granulocytes were capable of proliferating by autonomous division following infection and thus maintained their number in the hemocoel. Moreover, we showed that granulocyte phagocytosis is associated with autophagy and the activation of autophagy could be an efficient way to eliminate pathogens. Finally, the high accumulation of autophagic vacuoles in granulocytes leads to autophagic cell death (ACD).

## Materials and Methods

### Insects

The white-spotted flower chafers, *Protaetia brevitarsis seulensis* (Kolbe) were reared and maintained as previously described [35]. Briefly, larvae were reared in a constant environment (CE) incubator (MIR-553; Sanyo Electric Biomedical, Japan) at 25±1°C, 40–60% relative humidity, and a long photoperiod of 16 h light: 8 h dark cycle under aseptic conditions using well-fermented sterile oakwood sawdust.

### Preparation, identification, and counting of hemocytes

Hemolymph samples (approximately 1 ml) were withdrawn from the dorsal blood vessel with a sterile glass Pasteur pipette. Before collecting the hemolymph samples, each larva was cold-anaesthetized at −20°C for 5 min, surface-sterilized with 70% alcohol for a few seconds and rinsed with sterile water. About 1 ml of hemolymph was collected in a sterile Eppendorf tube in the presence (v/v) of anti-coagulant solution (98 mM NaOH, 186 mM NaCl, 17 mM EDTA, and 41 mM citric acid, (pH 4.5)), and mixed thoroughly. The hemolymph samples were centrifuged at 1000 g for 10 min at 4°C and the pellet was washed with anti-coagulant solution and used for in vitro experiments and hemocyte slide preparation. For the differential hemocyte counts (DHC), hemocytes were immediately placed in a sterile disposable hemocytometer slide (Neubauer Improved, iNCYTO C-Chip DHC-N01. www.incyto.com) (10 µl capacity). The number of each of the six types of hemocytes was counted in four squares using a light microscope (Leica DMI 3000B; 40× objective) and the percentages were determined per individual larva. The DHC were confirmed in three independent experiments at 4, 8, 12, 24, and 48 h post infection with bacteria and fungi, including five independent experiments for naïve larvae. 35 larvae (11,235 hemocytes) were used to determine the percentage of circulating hemocyte types, and the proportion of each cell population was expressed as the Mean (± SEM).

### Infection with potentially hazardous substances

The animals used in this study were always in the last larva instar. Larvae were cold-anesthetized and a finely pulled glass needle was shallowly inserted into the dorsal vessel; 40 µl of 2×10^7^ for *Escherichia coli*, 8×10^6^ for *Saccharomyces cerevisiae*, or 2.28×10^6^ carboxylate-modified polystyrene latex beads were injected into the hemocoel. For bacterial and fungal infection, green florescent protein (GFP)-expressing *E. coli* (modified DH5α) and *S. cerevisiae* (GFP-tagged *YDR385W*, Invitrogen, San Diego, CA) were incubated overnight at 37°C in Luria-Bertani’s rich nutrient medium (LB broth) and yeast extract-peptone-dextrose (YPD), and cultures were normalized to OD_600_ = 4 for bacteria and OD_600_ = 4 for yeast using a spectrophotometer (Eppendorf AG, Hamburg, Germany) prior to being injected into the larvae. The GFP-fluorescent or non-fluorescent carboxylate-modified polystyrene latex beads were 1 µm in diameter (aqueous suspension, 10% solids content-Sigma), diluted 1∶10 in 0.15 M sterile phosphate buffered saline (PBS), and 30 µl of beads were injected with a finely pulled glass needle. To explore the competence of phagocytes in vivo, cells of considerably larger size than the hemocytes in this insect, such as hemocytes of the Stag Beetle *Lucanus maculifemoratus* (Coleoptera**:** Lucanidae) and human breast cells (MCF10) were injected into the hemocoel (2×10^7^ cells in a volume of 30 µl). Next, hemocytes were collected and analyzed by direct puncture of the dorsal blood vessel of each larva at 4, 8, 12, 24, 48, 72, and 96 h post injection to evaluate hemocyte morphology, including phagocytosis and encapsulation and the relative quantity of the circulating hemocytes. Experiments were replicated using independently collected samples of hemocytes.

### Hemocyte labeling and visualization

Hemocyte visualization and counts were performed in naïve and challenged larvae (injected with potentially hazardous substances). Briefly, hemocytes were fixed with cooled paraformaldehyde (4%) in PBS pH 6.5 for 15 min and stained with DAPI (4′-6-diamidino-2phenylindole; 5 µg/ml) and fluorescently-conjugated phalloidin (F-actin cytoskeleton) (6.6 µM; Molecular probes) for 30 min. To label lysosomes, hemocytes were stained with the acidotropic dye LysoTracker Red (7.5 nM; Molecular Probes) for 30 min, washed three times with PBS, fixed with 4% paraformaldehyde for 15 min, washed again three times with PBS, and mounted. The red lysosomal fluorescence of 10,000 hemocytes per sample was determined by flow cytometry using the FL3 channel. To label hemocytes of the Stag Beetle *L. maculifemoratus,* CellTracker Green 5-chloromethylfluorescein diacetate (CellTracker™ Green CMFDA; Molecular Probes, Invitrogen Detection Technologies) was used as a fluorescent probe. Approximately 0.5 ml hemolymph samples (1×10^5^ cells/ml) were withdrawn from the dorsal blood vessel and collected in a sterile Eppendorf tube in the presence (v/v) of anti-coagulant solution. Then, CellTracker Green working solution (25 µM) was added in the tube, incubated for 30 min at 37°C, and injected into the hemocoel. At 12 h post injection, differential interferences contrast (DIC) and confocal microscopes were used for analysis. To assess the autophagosomes in GRs, the mammalian autophagy protein, LC3, was used as a marker. A cyto-ID® Autophagy Detection Kit (Enzo) was used to monitor the autophagic vacuoles in granulocytes according to the manufacturer’s protocol. Changes in relative percentages of autophagy were analyzed by flow cytometry at 0, 4, 8, 12, 24, 48, and 96 h post injection. Phenoloxidase activity (PO) staining was performed by fixing the hemocytes in 4% paraformaldehyde in PBS for 10 min, rinsing with PBS, and permeabilizing in 0.01% Triton X-100. After rinsing in PBS, the hemocytes were incubated with 2 mg/ml L-dopamine in 35% alcohol for 3 h. Hemocyte mitosis was directly observed using DAPI nuclear staining and measured by flow cytometry.

### Microscopy, image processing, and statistical analysis

All samples were observed with Leica DM2500 upright and Leica DMI 3000B inverted fluorescence microscopes. Images of whole hemocyte morphology, DAPI/fluorescently-conjugated phalloidin, LysoTracker, GFP CellTracker, and GFP-LC3 staining were acquired with a Leica photo camera (2048×1536 pixels resolution) using the LMD application software version 4.1. The confocal microscope images were taken and analyzed with the aid of an Olympus FV1000 confocal microscope and Olympus image application software. Statistical tests were performed using the Minitab software package. Significant difference of means was calculated using Student’s two-tailed t-test or one-way ANOVA at a probability (P) value of less than 5%.

### Fluorescence-activated Cell Sorting (FACS) Analysis

Hemocytes were analyzed and sorted on a BD™ FACSCanto flow cytometer (BD Bioscience; San Jose, CA) and sample analysis was performed according to protocols developed for this application using FACSDiva software from BD Biosciences. Green (GFP and fluorescein) and red (Propidium iodide (PI) and rodamine) fluorescence were detected in the FL1 (530/30 band-pass) and FL3 (610/20 band-pass) instrument parameters, respectively. Apoptotic and necrotic cells were distinguished by using the annexin-V/Propidium Iodide (PI) kit according to the manufacturer’s instructions (Roche Diagnostics). After washing in PBS, hemocytes were re-suspended for 10 min in the staining solution and analyzed by flow cytometry. The percentages of apoptotic and necrotic cells were determined from 10,000 hemocytes per sample, by using the FL1 channel for annexin-V and the FL2 channel for PI.
